# Bibliometric analysis and visualization of research trends on HIV-1 capsid inhibitors (2000–2022)

**DOI:** 10.3389/fphar.2023.1282090

**Published:** 2023-10-23

**Authors:** Lin Sun, Tongchao Zhang, Shujing Xu, Xujie Zhang, Peng Zhan, Xinyong Liu

**Affiliations:** ^1^ Department of Pharmacy, Qilu Hospital of Shandong University, Jinan, Shandong, China; ^2^ Key Laboratory of Chemical Biology (Ministry of Education), Department of Medicinal Chemistry, School of Pharmaceutical Sciences, Shandong University, Jinan, Shandong, China; ^3^ Clinical Research Center of Shandong University, Clinical Epidemiology Unit, Qilu Hospital of Shandong University, Jinan, Shandong, China

**Keywords:** HIV-1, capsid inhibitor, WoSCC, bibliometrics, VOSviewer

## Abstract

**Background:** Acquired immunodeficiency syndrome (AIDS) has seriously endangered human life and health, the main pathogenic agent is human immunodeficiency virus type 1 (HIV-1). The combination antiretroviral therapy (cART) has shown serious drug resistance and side effects, and the discovery of HIV-1 capsid inhibitors is an effective way to solve the problem. Recent studies have shown significant progress in the research of HIV-1 capsid inhibitors. However, there is still a lack of comprehensive overview of bibliometric analysis in this field. This study aimed to provide the research trends and hotspots of HIV-1 capsid inhibitors.

**Method:** Publications related to HIV-1 capsid inhibitors from 2000 to 2022 were searched on the Web of Science Core Collection (WoSCC) database and screened according to inclusion criteria. VOSviewer was conducted to evaluate the results.

**Results:** 96 publications from 25 countries were finally included, and the number of annual publications related to HIV-1 capsid inhibitors showed an increasing trend. The United States was the most productive country with the most publication number, H-index, and total citation number, as well as the widest international cooperation. The most popular journal in this field was *Journal of Virology*. Drexel University was the most productive institution, and Simon Cocklin participated in the most publications. Keywords co-occurrence analysis exhibited that studying the molecular mechanism of capsid protein, discovering drug candidates, and improving antiretroviral therapy are the main and hot topics in this field.

**Conclusion:** This is the first bibliometric study in the field of HIV-1 capsid inhibitors, which comprehensively analyzed the research trends and hotspots in this direction. This work is expected to provide the scientific community with new insights to promote the research of HIV-1 capsid inhibitors.

## 1 Introduction

HIV is an infectious retrovirus that primarily targets immune cells such as CD4^+^T lymphocytes and macrophages. The final stage of HIV infection is the emergence of AIDS (Turner and Summers, 1999). Since the first case of AIDS in the United States was reported in 1981 to the end of 2022, about 39 million people are living with HIV in the world, and HIV is still one of the biggest public health challenges (World Health Organization, 2022). The main pathogenic subtype of HIV is HIV-1 (Freed and Mouland, 2006).

In view of the characteristics of HIV-1, such as high replication and high variation, single drug treatment can cause drug resistance. Therefore, three or more drugs acting on different targets are generally used together in clinical practice as cART (Castro and Camarasa, 2018). The emergence of cART method has greatly improved the living quality of patients, making AIDS from a fatal disease to a chronic controllable disease (Palmisano and Vella, 2011). However, cART can not completely eliminate HIV in the body and the treatment is expensive. Long-term use of cART may cause serious side effects (Murphy et al., 2001; Feinstein et al., 2015). Therefore, developing efficient and low-toxicity anti-HIV-1 drugs with unique mechanisms of action is an effective way to overcome existing drug resistance challenges and enrich clinical treatment regimens.

Capsid protein (CA) plays a crucial role in the HIV-1 lifecycle and is highly conserved, thus gradually becoming a research hotspot for novel HIV-1 inhibitors. Small molecule inhibitors can bind to multiple binding sites on capsid protein and induce or disturb protein-protein interactions and conformational changes. Capsid protein participates in different protein interactions at different stages of the lifecycle, which makes resistance selection complicated. Therefore, the assembly and disassembly of HIV-1 capsid become highly sensitive processes that can deliver new generations of antivirals. However, even though hundreds of studies on HIV-1 capsid inhibitors have been published since the beginning of this century, a comprehensive overview of bibliometrics is still lacking so far.

Bibliometrics is a calculation and statistics method based on the literature database to quantitatively and qualitatively analyze the publications in a particular research direction (Tran et al., 2018). Bibliometrics can not only provide an evaluation of previous studies but also provide research directions for future (Zhai et al., 2018; Zhan et al., 2020). Bibliometrics has become one of the commonly used techniques for evaluating the credibility, quality, and impact of academic work (Ellegaard and Wallin, 2015; Rondanelli et al., 2016). Accordingly, this study aimed to conduct a bibliometric analysis of articles on HIV-1 capsid inhibitors (published from 2000 to 2022) to obtain the research status and major contributors, besides, the research trends and development hotspots in this field were prospected.

## 2 Methods

### 2.1 Search strategy

WoSCC (https://www.webofscience.com/wos/woscc/basic-search), one of the most influential and largest scientific citation index databases worldwide, was used in this study to perform literature search on 12 April 2023. As it has comprehensive academic information resources, the WoSCC database is applied frequently in bibliometric analysis, which ensures the representativeness and accessibility of literature (Shen et al., 2018; Shen et al., 2019; Zhang et al., 2020). The search formula is TS = {[(Human Immunodeficiency Virus*) OR (HIV*) OR (AIDS Virus*) OR (Acquired Immune Deficiency Syndrome Virus*) OR (Acquired Immunodeficiency Syndrome Virus*) AND ((CA) OR (Capsid Protein*)) AND ((Inhibitor*) OR (Modulator*)]}. A total of 827 records were obtained.

### 2.2 Data screening

**FIGURE 1:**
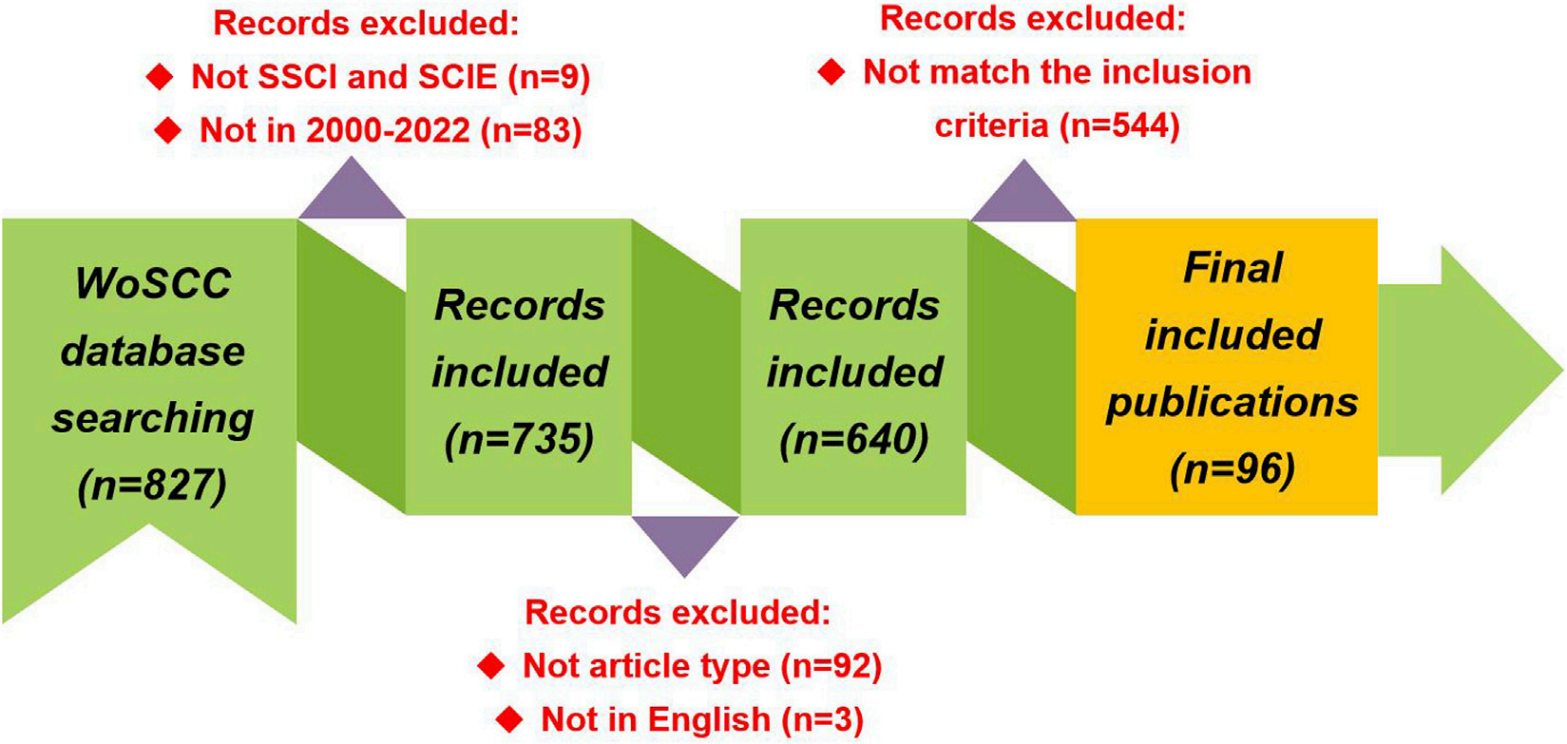
Publications screening flowchart.

As represented in Figure 1, further detailed screening criteria were imposed on the preliminary search results: 1) The editions of WoSCC selected Science Citation Index Expanded (SCIE) and Social Sciences Citation Index (SSCI) (9 records excluded), 2) The publication timespan was limited between 2000 and 2022 (83 records excluded), 3) The document type was set to “article” (92 records excluded), 4) The document language selected “English” (3 records excluded), and 5) The research content of included publications must be related to design, synthesis, activity evaluation, or mechanism of action study of HIV-1 capsid inhibitors (544 records excluded). The collection and analysis of data was completed within 1 day to minimize deviations caused by database updating. Finally, a total of 96 articles were included in the final analysis.

### 2.3 Data analysis

In this study, several analysis items of selected publications were introduced, including number of publications and citations, Hirsch index (H-index), impact factor (IF), contribution of countries, journals, institutions, and authors, as well as keywords co-occurrence, etc. Specifically, related indicators of countries, journals, institutions, and authors were counted and analyzed by inner Analyze Results and Citation Report modules of WoSCC and the bibliometric tool VOSviewer (version 1.6.18) (Ramin et al., 2016). The WPS Office S 2023 was used for quantitative analysis and plotting of the above data. Additionally, the co-authorship network and keyword co-occurrence were visualized by VOSviewer (Khalil and Gotway Crawford, 2015).

## 3 Results

### 3.1 Temporal distribution map of publications

**FIGURE 2:**
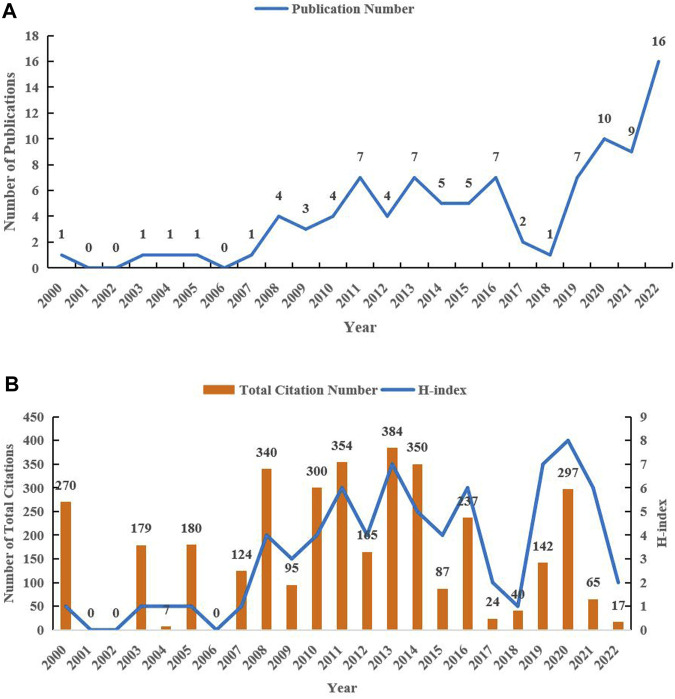
Temporal distribution map of publications in the field of HIV-1 capsid inhibitors from 2000 to 2022. **(A)** Annual number of the publications; **(B)** Annual total citations and H-index.

In this study, 96 articles were included for bibliometrics analysis. Figure 2A presented the annual number of publications. From 2000 to 2007, there were few articles published (the maximum number of articles published annually was 1). From 2008 to 2016, the annual number of publications increased and remained relatively stable (with a maximum of 7 publications). Subsequently, from 2017 to 2018, the number of publications decreased significantly (with an annual maximum of 2 publications). From 2019 to 2022, the annual number of articles published showed a significant upward trend, reaching the highest value of 16 in 2022. The number of articles published over the past 4 years accounted for 44% of the total publications. As of the retrieval date, 96 publications have been cited 3657 times, with an average of 38.09 times. As depicted in Figure 2B, the years with high citations (>300 times) were 2008 (340 times), 2010 (300 times), 2011 (354 times), 2013 (384 times), and 2014 (350 times), respectively. Despite having the highest number of articles published in 2022, the number of citations was very low (17 times), possibly due to the proximity of the data collection time. Figure 2B also revealed the H-index (defined as the number of papers with citation number ≥ h) of articles published annually, and the year with the highest H-index was 2020 (8).

### 3.2 Distribution of productive countries

**FIGURE 3:**
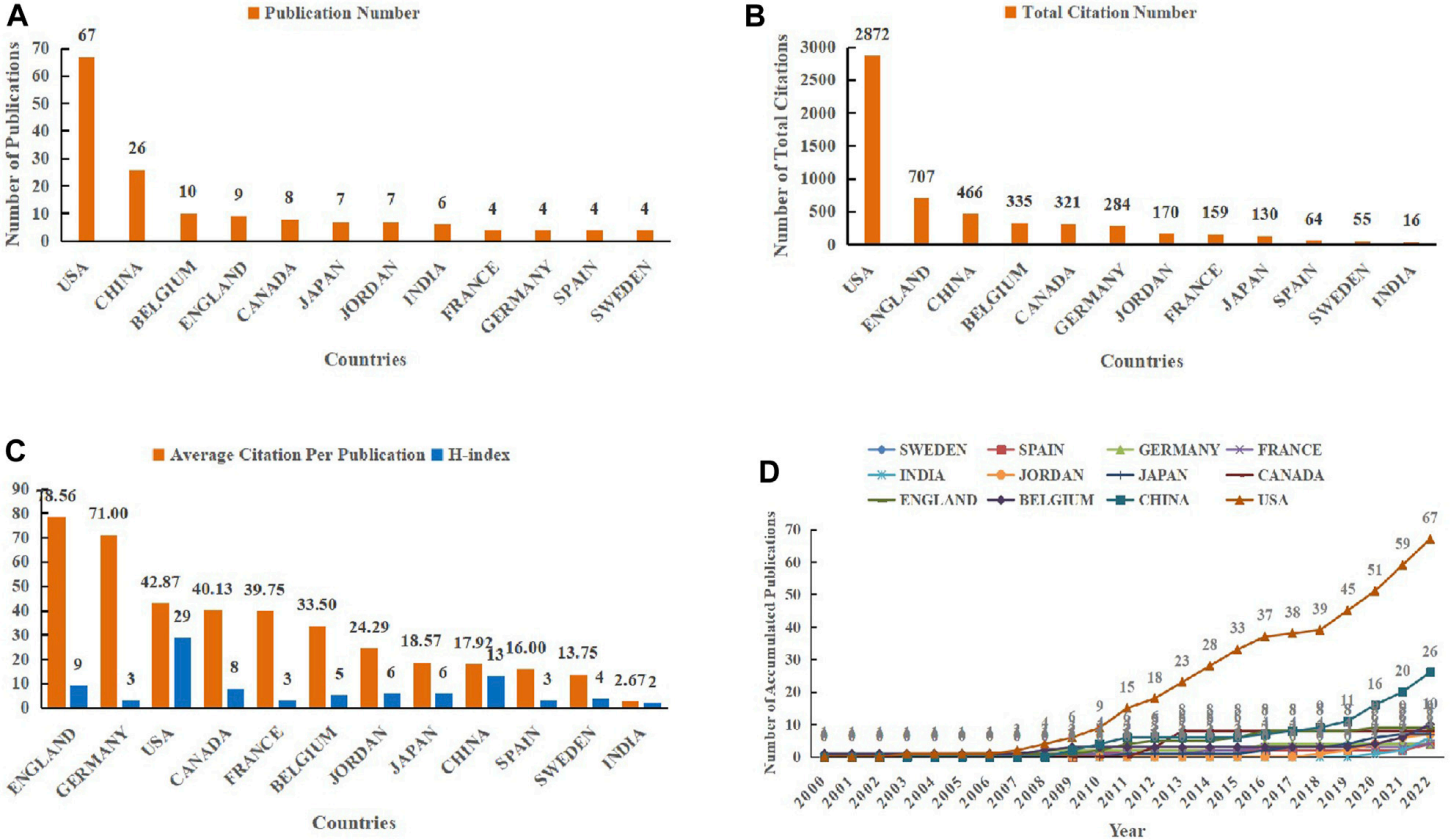
Top 12 productive countries in the field of HIV-1 capsid inhibitors from 2000 to 2022. **(A)** Number of publications; **(B)** Number of total citations; **(C)** Average citations and H-index; **(D)** Accumulated number of articles published in countries.

In this study, 25 countries have participated in the 96 included publications. As the most productive country, the United States has participated in 67 articles, followed by China (26), Belgium (10), England (9), Canada (8), Japan (7), Jordan (7), India (6), France (4), Germany (4), Spain (4), and Sweden (4), etc. (Figure 3A). As revealed in Figure 3B, the top 3 countries in terms of total citations were the United States (2,872 times), England (707 times), and China (466 times), respectively. However, in the ranking of average citations, England (78.56 times) rose to the first place, followed by Germany (71.00 times), and the United States (42.87 times). Next, the United States (29) once again ranked top 1 in H-index among all countries, followed by China (13), England (9), Canada (8), Japan (6), Jordan (6), etc. (Figure 3C). The number of cumulative published articles in countries was also depicted in Figure 3D, it is evident that the growth trend of the United States was the fastest, continuing from 2007 to 2016, then entering the platform period, followed by a sharp increase in total publications in 2018, and maintaining a growth momentum throughout the survey period until 2022. Another country with outstanding growth momentum was China, after experiencing a slow growth platform period from 2011 to 2017, the cumulative number of articles published began to surge, from 9 in 2018 to 26 in 2022.

**FIGURE 4:**
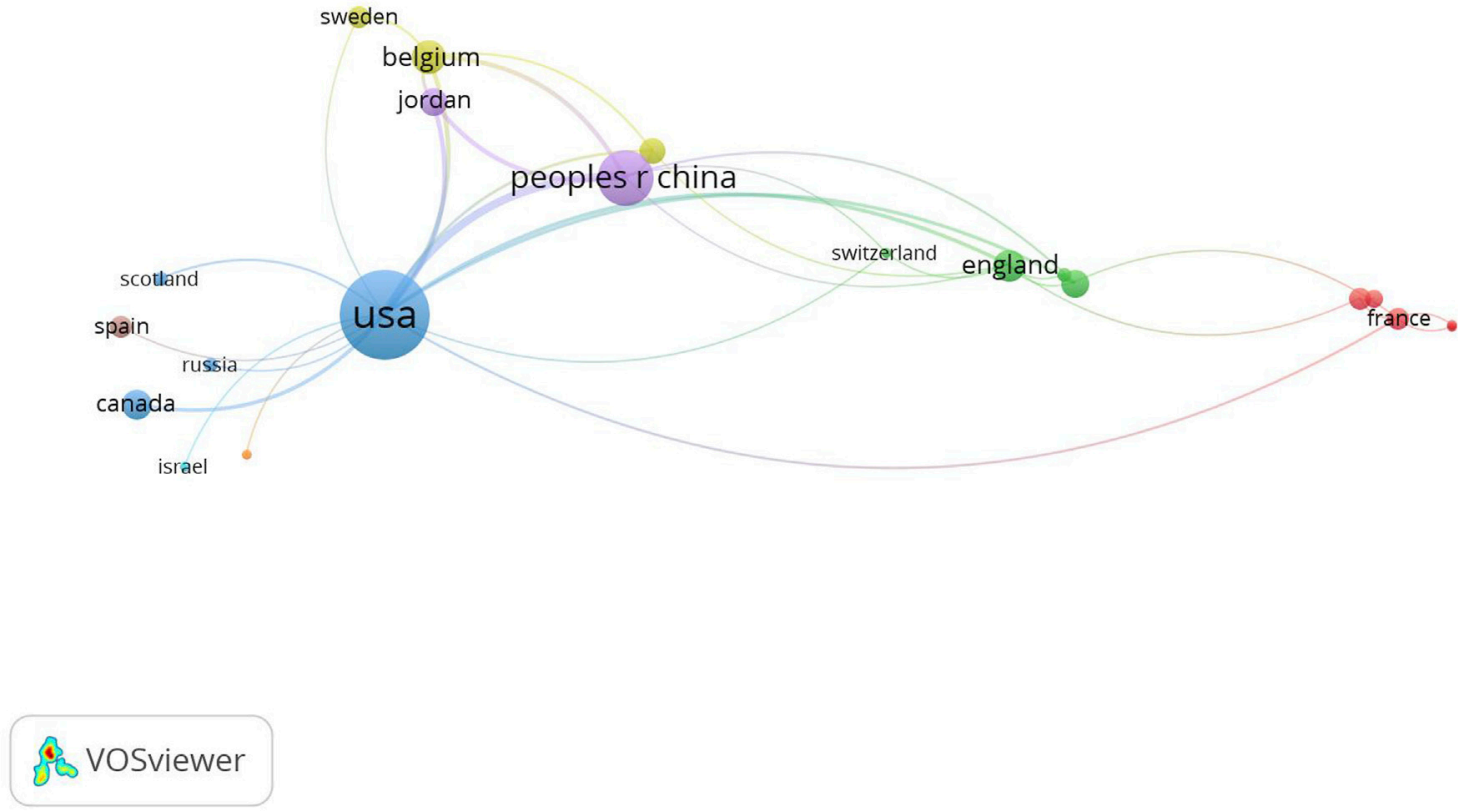
Cooperation map between countries. Each node represented a country. The size of the node represented the number of publications, and the thickness of links between nodes represented the collaboration intensity.

To display the cooperation map of countries, the co-authorship analysis in VOSviewer was performed in this study, and the parameter minimum number of documents of a country was set as 1, all 25 countries met the threshold. Figure 4 revealed the intensity of cooperation between countries in the field of HIV-1 capsid inhibitors, the United States showed the most extensive international cooperation, among which it had the closest ties with China. In addition, China also cooperated more closely with the United States, followed by Belgium, Jordan, etc. Figure 4 indicated that the United States was in an absolute leading position in the current field, but over time, other countries represented by China were striving to catch up and narrow the gap with it.

### 3.3 Distribution of productive journals, institutions, and authors

**FIGURE 5:**
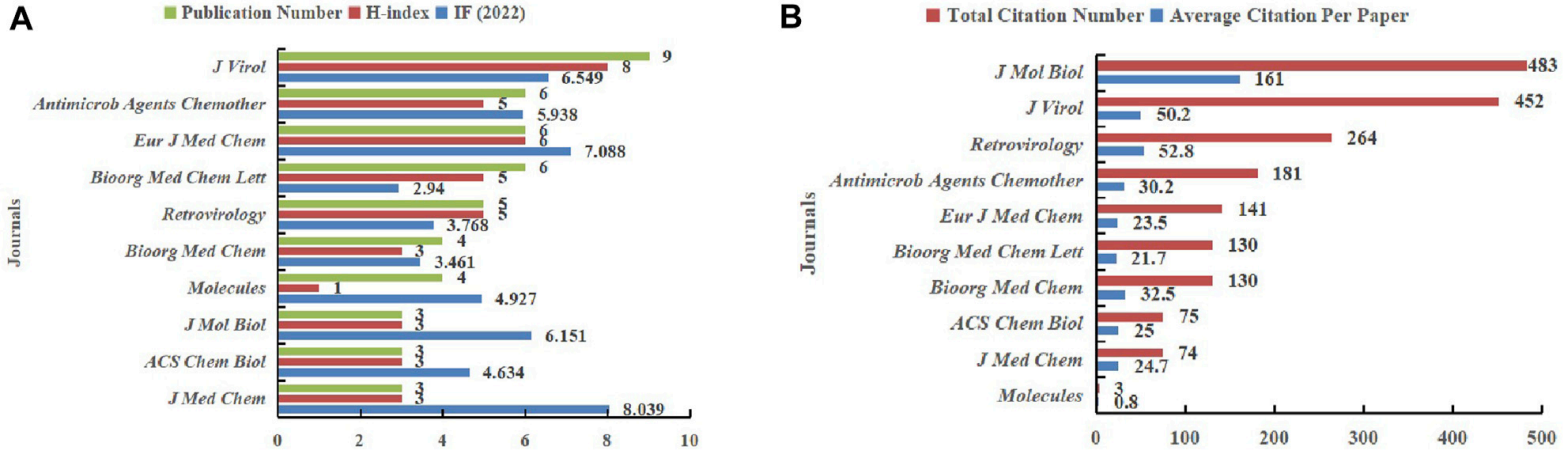
Top 10 journals in the field of HIV-1 capsid inhibitors from 2000 to 2022. **(A)** Number of publications, H-index, and IF (2022); **(B)** Number of total citations, and average citations per publication.

96 included articles were from 48 journals, with 49 articles published in the top 10 productive journals, accounting for 51% of the total. Figure 5 provided detailed information on the top 10 journals. *Journal of Virology* ranked first with 9 articles published, followed by *Antimicrobial Agents and Chemotherapy* (6), *Bioorganic Medicinal Chemistry Letters* (6), *European Journal of Medicinal Chemistry* (6), *Retrovirology* (5), *Bioorganic Medicinal Chemistry* (4), *Molecules* (4), *ACS Chemical Biology* (3), *Journal of Medicinal Chemistry* (3), and *Journal of Molecular Biology* (3) (Figure 5A). In terms of total citations, the top 3 journals in the rankings were *Journal of Molecular Biology* (483 times), *Journal of Virology* (452 times), *Retrovirology* (264 times), respectively (Figure 5B). In the item of average citations, *Journal of Molecular Biology* once again ranked first with 161 times, followed by *Retrovirology* (52.8 times) and *Journal of Virology* (50.2 times) (Figure 5B), while the H-index of *Journal of Virology* (8) was the highest (Figure 5A). We also listed the IF of each journal in 2022, with the top 3 being *Journal of Medicinal Chemistry* (8.039), *European Journal of Medicinal Chemistry* (7.088), and *Journal of Virology* (6.549) (Figure 5A).

**FIGURE 6:**
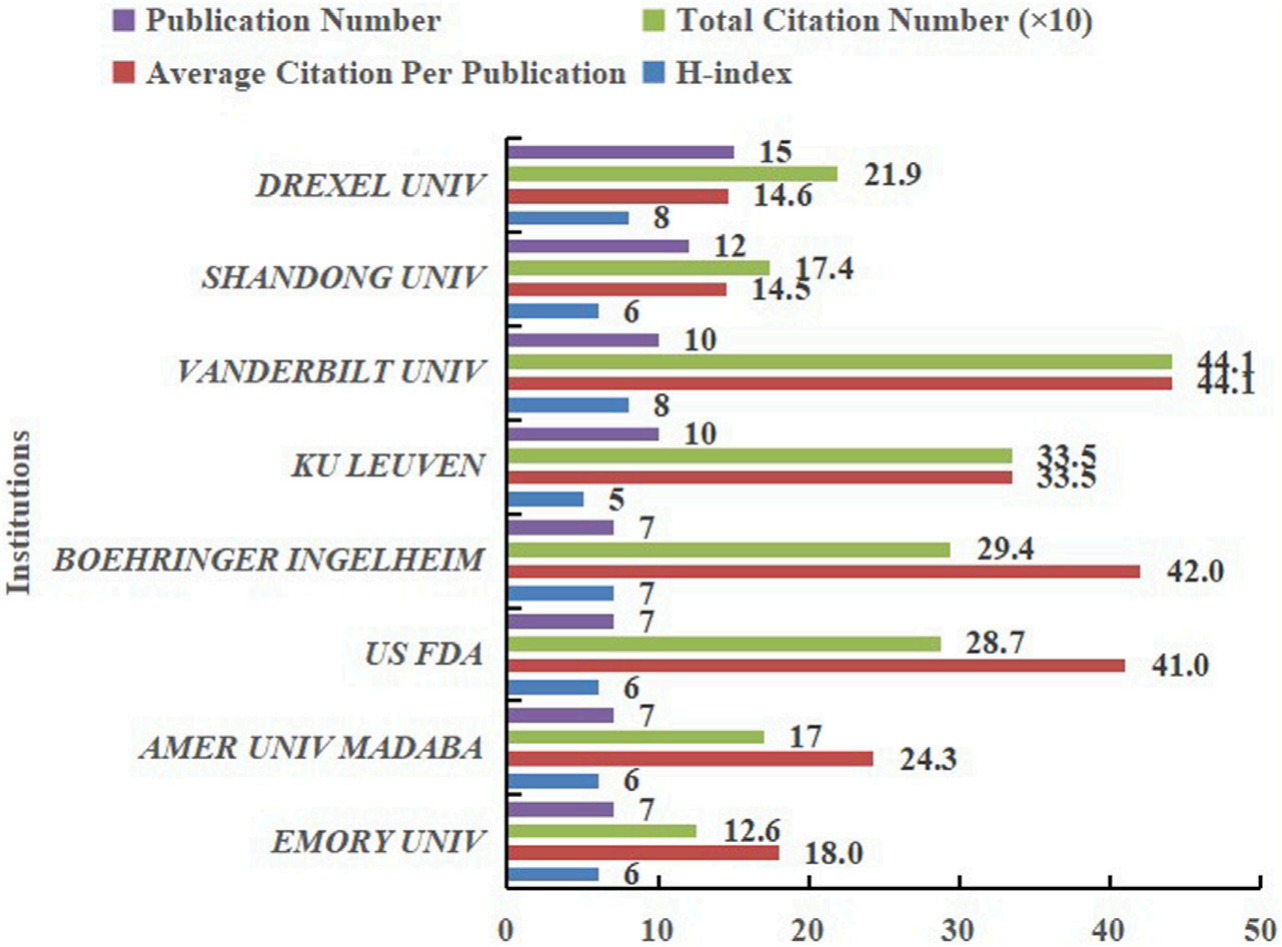
Contributions of top 8 productive institutions in the field of HIV-1 capsid inhibitors from 2000 to 2022: Number of publications, total citations, average citations per publication, and H-index.

Figure 6 demonstrated the top 8 productive institutions with published articles, as well as the total citations, average citations, and H-index. Drexel University has published 15 articles, ranking first, followed by Shandong University (12), Vanderbilt University (10), KU Leuven (10), etc. However, the institution with the highest total citations was Vanderbilt University (441 times), and its average citations also ranked first (44.1 times). In addition, Vanderbilt University and Drexel University have the same H-index (8) and both ranked first among all institutions.

VOSviewer showed that 529 authors participated in the included articles, and to screen out the productive authors, co-authorship analysis was conducted: set a minimum of 5 publications and a minimum of 100 citations, resulting in a total of 27 core authors being included. Simon Cocklin, who has published 15 papers, was the most productive researcher, followed by Xinyong Liu (12), Peng Zhan (12), Dick Alexej (12), Christopher Aiken (10), and others (Table 1). Nevertheless, Christopher Aiken was the author with the most total citations (443 times), with average citations also ranked first (44.3 times) (Table 1).

**TABLE 1:** Top 8 productive authors in the field of HIV-1 capsid inhibitors from 2000 to 2022.

Rank	Author	Documents	Total citations	Average citations per paper
1	Simon Cocklin	15	225	15
2	Xinyong Liu	12	180	15
3	Peng Zhan	12	180	15
4	Dick Alexej	12	149	12.4
5	Christopher Aiken	10	443	44.3
6	Megan E. Meuser	8	187	23.4
7	Lin Sun	8	136	17
8	Dongwei Kang	8	127	15.9

**FIGURE 7:**
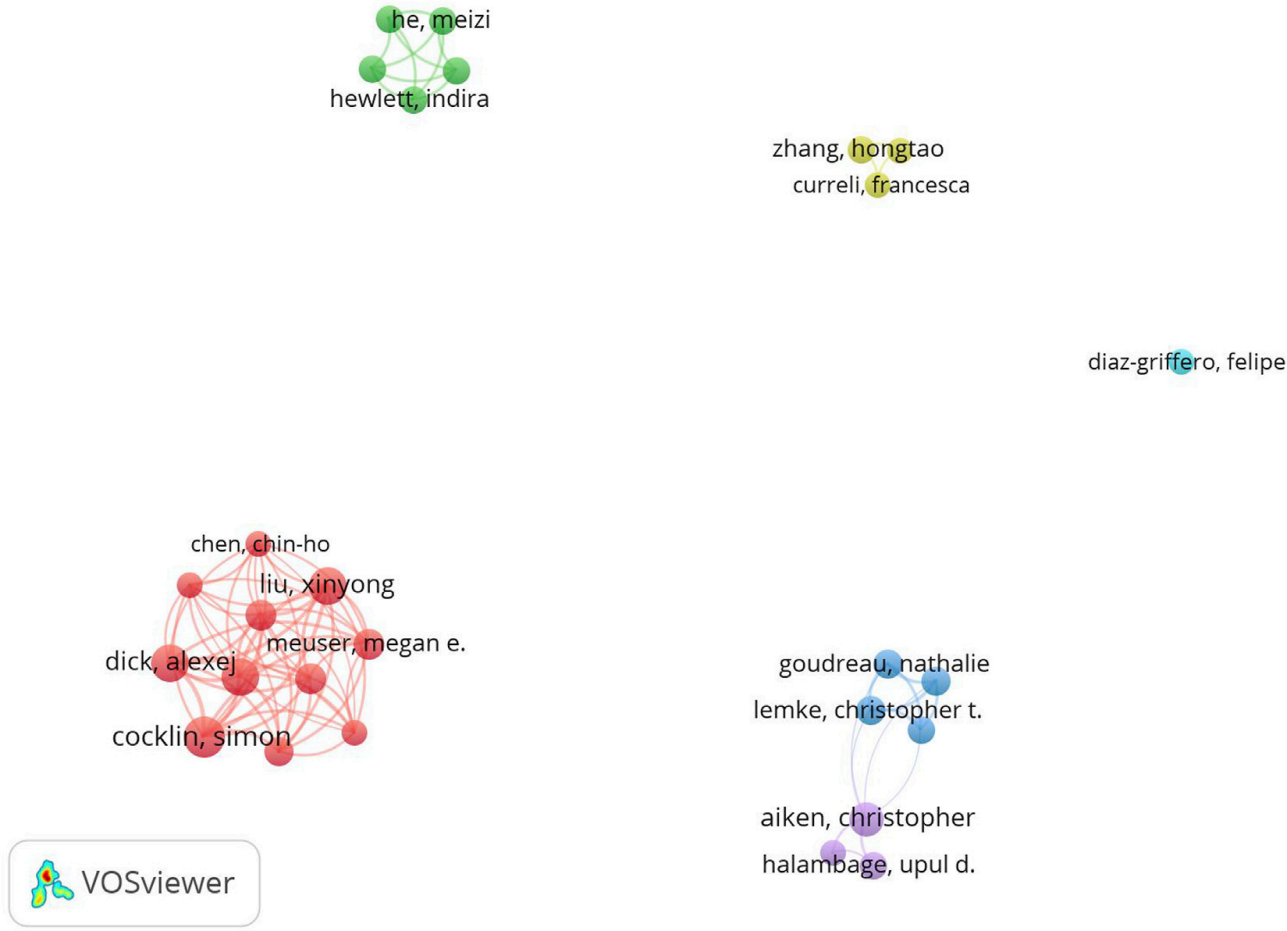
Co-authorship analysis among 27 core authors. Each node represented an author, the size of the node represented the number of published articles, different clusters were marked with different colors, and links between nodes represented collaborative relationships.

As shown in Figure 7, 27 core authors formed 6 clusters, with the largest being the red cluster represented by Xinyong Liu and Simon Cocklin. However, apart from the blue and purple clusters that cooperated closely, there was a lack of cooperation among the others.

### 3.4 Network visualization of keywords co-occurrence

Network visualization analysis of keyword co-occurrence is an effective way to provide insights into the most popular directions in certain research field, and VOSviewer software is one of the most important tools for performing keyword analysis (van Eck and Waltman, 2010). To get a better perspective, we first have made slight adjustments to some keywords: for example, unify CA protein, capsid as capsid protein, and human-immunodeficiency-virus, immunodeficiency-virus type-1 as hiv-1, and so on.

After that, we set the minimum frequency of keyword occurrences to 5, and out of all 420 keywords, a total of 41 core keywords were introduced into the analysis.

**FIGURE 8:**
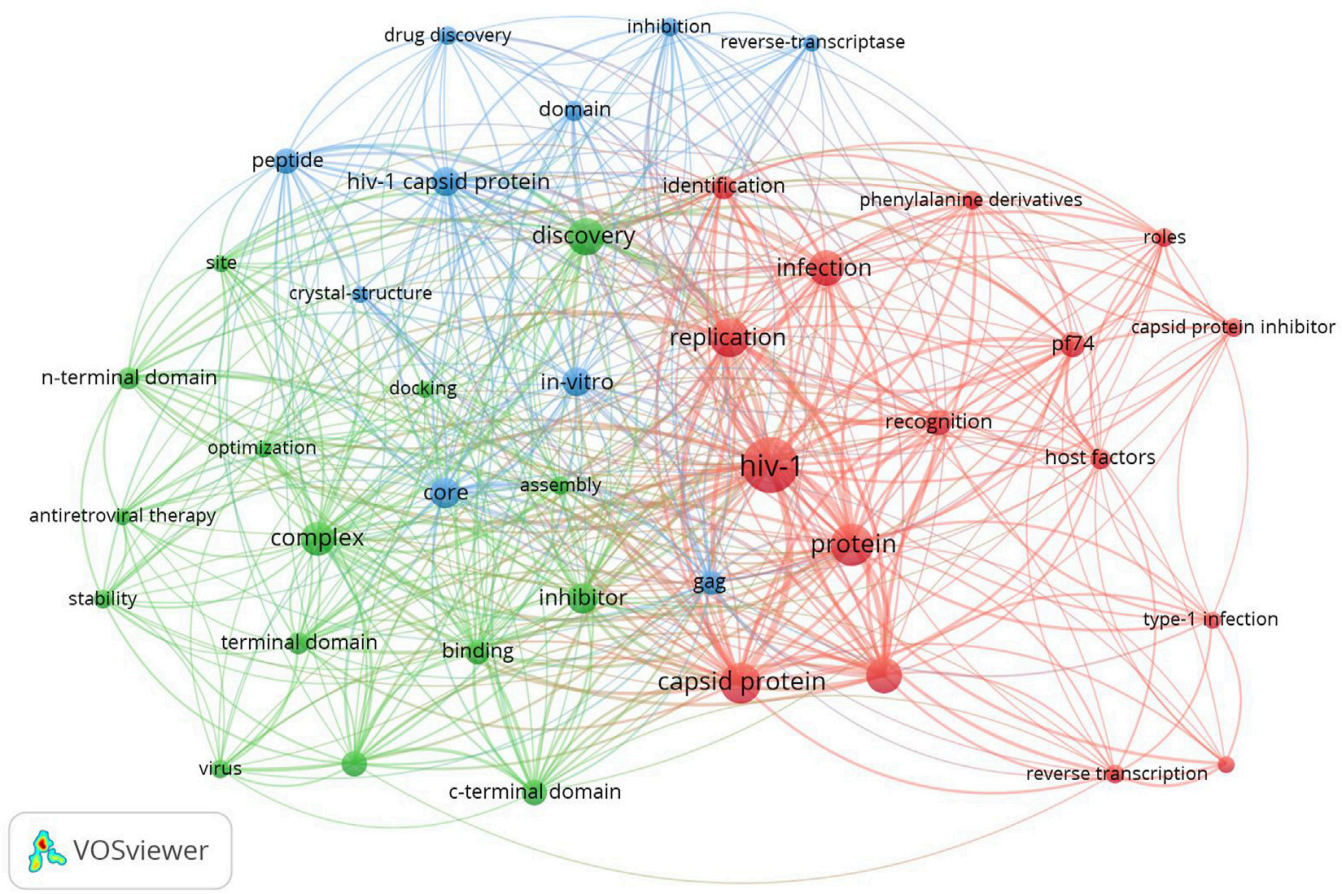
Network visualization of keywords co-occurrence in the field of HIV-1 capsid inhibitors from 2000 to 2022. Each node represented a keyword and node size indicated the occurrence frequency of the keyword, different clusters were painted with different colors and the links represented the keywords co-occurrence.

The total link strength of each core keyword was calculated based on VOSviewer to create a network visualization map, and 3 clusters were found (Figure 8): 1) The red cluster corresponded to the terms searched most frequently (16 items) and the keywords represented including HIV-1, capsid protein, and replication. 2) The green cluster includes the second most frequently searched terms (15 items) and the keywords represented including discovery, complex, and inhibitor. 3) Finally, the keywords with the lowest search frequency made up the blue cluster (10 items), and the keywords represented including core, *in-vitro*, and gag.

## 4 Discussion

In this study, we have explored the research trends and hotspots in the field of HIV-1 capsid inhibitors from 2000 to 2022 with the bibliometrics software. The analysis found that the number of articles published annually has shown a sustained growth trend over time, reaching its peak in 2022. In 2000, only one article was included and cited 270 times, making it the year with the highest average citation, indicating that this article has a very important enlightening role for the research direction and it is necessary to continue to pay attention to the author’s academic output in this field (De Clercq, 2000). In addition, the articles published in 2013 have the highest total citations and the second highest H-index, which indicated that certain research progress has been made in this field this year, and therefore more attention has been paid. The inconsistency between the number of citations and the number of publications in 2021 and 2022 may be due to their proximity to the data collection time of this study and insufficient citations. Undoubtedly, the citation frequency of articles in the past 2 years will significantly increase over time.

This study also investigated the publication number of various countries within the set period of time, with the United States, China, and Belgium ranking in the top 3 respectively. It is worth mentioning that the number of papers participated by the United States is 67, which is equal to the sum of the second to seventh places, and its H-index is also far ahead. Meanwhile, the total citation number of articles participated by the United States also ranked among the top, even surpassing the sum of other countries. In addition, the United States maintained the widest and deepest international cooperation with others. In terms of the cumulative number of publications, China ranked second, which is particularly noteworthy. Publications of China have increased dramatically since 2018, and high-quality research has also improved accordingly. The above information reflected the absolute leading position of the United States in this research field, while other countries represented by China were also striving to catch up and gradually narrow the gap.

There are 8 institutions that have published more than 7 articles, of which 4 are from the United States, with one each from China, Belgium, Germany, and Jordan, reflecting the mature scientific research capabilities of the United States. Drexel University ranked first in terms of publication number, indicating its high research level in this field, and can be considered as a key institution for academic cooperation and exchange. More than half of the articles were published in 12 core journals, which can be divided into four categories: virology, therapeutics, medicinal chemistry, and molecular biology. Among them, *Journal of Virology* exhibited the highest publication number and H-index, indicating that this publication possessed a high academic recognition. Continuously pay attention to these 12 core journals to obtain high-quality latest research progress in this field. In the research field of HIV-1 capsid inhibitors, major research teams represented by Simon Cocklin, Aiken Christopher, and others have close internal connections but lack external communication. Academic cooperation can be attempted to achieve strong alliances and make further breakthroughs in this field.

The visualization network of keywords co-occurrence revealed that core keywords were mainly divided into 3 clusters: (1) HIV-1 replication and capsid protein, (2) structural biology of HIV-1 capsid and inhibitor optimization, (3) drug discovery and *in vitro* evaluation, reflecting the current focus of this field, future research on HIV-1 casid inhibitors will be more diverse and in-depth to achieve breakthrough results. “HIV-1” and “capsid protein” were the keywords with the highest frequency, and closely related to “inhibitor”, “host factors”, “terminal domain”, “reverse transcription”, “stability”, “assembly”, etc., and the research based on molecular docking and the crystal structure has also greatly promoted the drug discovery.

HIV-1 capsid protein is a structural protein necessary for the formation of an infectious mature virus particle, which is wrapped with viral genes and key enzymes essential for virus replication. HIV-1 capsid plays a crucial regulatory role in both the early (uncoating, reverse transcription, nuclear import, and integration) and late (translation, assembly, maturation) stages of virus replication. Research has shown that if the formation process of the conical lattice shell is disrupted, the newly formed HIV-1 particles lose their infectivity (Yamashita and Engelman, 2017; Engelman and Singh, 2018). The normal assembly and structural stability of capsid protein are crucial for the infectivity of viruses, making capsid a new and hot target for anti-HIV-1 drug research (Campbell and Hope, 2015; Thenin-Houssier and Valente, 2016).

The mature capsid is a conical fullerene-like lattice shell, and each lattice contains about 1500 capsid monomers, including 250 hexamers and 12 pentamers (Engelman, 2021). Capsid monomer is composed of N-terminal domain (NTD), C-terminal domain (CTD), and a flexible linker in the middle. At present, there are at least three binding sites of CA with several kinds of inhibitors on NTD, CTD, and NTD-CTD interface between adjacent monomers of capsid hexamers. The co-crystal structure indicated that compound PF74 binds to hydrophobic pockets on the NTD surface of capsid hexamer subunit and interacts with the CTD of the adjacent subunit (Bhattacharya et al., 2014; Saito et al., 2016). Gilead conducted extensive structural modifications on PF74 and ultimately discovered the drug candidate GS-6207 (Yant et al., 2019). Research has shown that GS-6207 exhibited strong *in vitro* antiviral activity (0.02–0.16 nM) and has synergistic antiviral effects (Margot et al., 2021). The low clearance rate, moderate distribution volume, and long half-life (15–38 h) of GS-6207 indicated that the drug has long-term pharmacokinetic properties, and can still exhibit excellent antiviral effects even when administered every 6 months (Carnes et al., 2018; Flexner et al., 2021). GS-6207 has been approved by the FDA for the treatment of multidrug-resistant HIV-1 as part of the antiretroviral therapy regimen, and this approval is supported by the results of the CAPELLA study (NCT04150068).

Although finding an inhibitor with strong anti-HIV activity is still a major challenge, the extensive application of medicinal chemistry strategies will greatly improve the efficiency and success rate of drug research and development (Wu et al., 2019; Xu et al., 2020; Sun et al., 2021; Ding et al., 2023; Zhao et al., 2023). For example, in the discovery of lead compounds, high-throughput screening, pharmacophore model-guided screening, drug repositioning, and other strategies can be used. In the optimization of inhibitors, multiple strategies such as bioisosterism replacement, scaffold hopping, molecular hybridization, dual inhibitors, covalent inhibitors, conformational restriction, multivalent agents, etc. can be applied simultaneously.

In addition to discovering small molecule inhibitors, developing safe and effective HIV vaccines (Wen and Sun, 2020), HIV CRISPR/Cas9 gene editing (Xiao et al., 2019; Hashmat et al., 2020), and shock and kill (Deeks, 2012; Abner and Jordan, 2019) strategies are also important treatment methods in AIDS therapy. Besides, there are other therapeutic approaches that deserve attention, such as stem cell transplantation, broad-spectrum neutralizing antibody therapy, specific T cell therapy, and traditional Chinese medicine prevention and treatment. Scientists are constantly trying to explore new HIV treatment methods to find the possibility of a complete cure for HIV.

## 5 Conclusion

This article specifically studied the publication status, keyword co-occurrence, keyword clustering, and core authors and journals related to HIV-1 capsid inhibitors from 2000 to 2022, clarifying the research hotspots and development trends in this field. The United States is in an absolute leading position, and other countries represented by China are catching up and gradually narrowing the gap. Due to the late start of capsid research, the previous studies mainly focused on the analysis of the microstructure and molecular mechanism of capsid protein, and the development of capsid inhibitors has certain blindness and contingency. With the development of molecular biology and computer technology, future methods based on co-crystal structure and computer-aided drug design will undoubtedly greatly promote the discovery of novel and efficient capsid inhibitors. The long-acting antiviral drug represented by GS-6207 will effectively improve the patients’ medication compliance and greatly inhibit the virus replication to improve the treatment effect, which is an important research direction of anti-AIDS drugs in the future.

This study also has some shortcomings that should be taken into account when interpreting research results. Firstly, the publications are only from SCIE and SSCI of the WoSCC database, which may lead to incomplete document retrieval. Notably, WoSCC has high-quality literature resources and the database can be continuously updated, which has been widely applied in bibliometric analysis (Shah et al., 2021; Zhang et al., 2021; Chen et al., 2022). Secondly, our analysis only included English literature, which may mean publications in other languages were underestimated. Finally, despite our in-depth analysis and interpretation of the results, there may still be subjective bias in the understanding of software analysis, which effects the objectivity of results.

## Data Availability

The original contributions presented in the study are included in the article/Supplementary material, further inquiries can be directed to the corresponding authors.
